# Human Plasma Levels of VEGF-A, VEGF-C, VEGF-D, their Soluble Receptor - VEGFR-2 and Applicability of these Parameters as Tumor Markers in the Diagnostics of Breast Cancer

**DOI:** 10.1007/s12253-018-0527-0

**Published:** 2018-11-01

**Authors:** Monika Zajkowska, Emilia Lubowicka, Wojciech Fiedorowicz, Maciej Szmitkowski, Jacek Jamiołkowski, Sławomir Ławicki

**Affiliations:** 1 Department of Biochemical Diagnostics, Medical University of Bialystok, Waszyngtona 15A, 15-269 Bialystok, Poland; 2 Department of Esthetic Medicine, Medical University of Bialystok, 15-267 Bialystok, Poland; 3 Department of Oncological Surgery, Maria Sklodowska-Curie Oncology Center, 15-001 Bialystok, Poland; 4 Department of Population Medicine and Civilization Diseases Prevention, Medical University of Bialystok, 15-269 Bialystok, Poland

**Keywords:** VEGF family members, Diagnostic utility, AUC, ROC

## Abstract

VEGF family members are important factors in promoting angio- and lymphangiogenesis. The aim of this study was to investigate concentrations, diagnostic utility and power of VEGF-A, VEGF-C, VEGF-D and VEGFR-2 in comparison to CA15–3 in breast cancer (BC) patients. The study included 120 BC patients and 60 control patients (28 with benign breast tumors and 32 healthy women). Plasma levels of tested parameters were determined by ELISA, CA15–3 by CMIA. Concentrations of all parameters showed statistical significance when compared BC patients to controls. VEGF-D showed the highest SE (82.50%) in total BC group. Highest SP and PPV in total BC group showed VEGF-A(76.67%;84.78%,respectively), but lower than CA15–3. Highest NPV showed VEGF-C(52.33%), but it was lower than CA15–3. VEGF-C was also the best parameter which had statistically significant AUC in total cancer group (0.7672), but also stages I(0.7684) and II(0.7772). In the total group of BC almost all tested parameters showed statistically significant AUC, but a maximum range was obtained for the combination of VEGF-C + CA15–3(0.8476). The combined analysis of tested parameters and CA15–3 resulted in increase in SE and AUC values, which provides hope for developing a new panel of biomarkers that may be used in the diagnosis of BC in the future.

## Introduction

In the United States, in 2015, cancers caused 22% of all documented deaths. It places tumors on the second position from all deaths in this country. Breast cancer (BC) is the most frequent cancer occuring in women worldwide [1, 2]. Only in this country, 266,120 new female cases and 40,920 female deaths are being estimated by American Cancer Society to occur in 2018 [3]. The most effective way to combat cancer is its prevention and early detection. Therefore, finding markers that would detect malignant cell transformation as early as possible is vital [4].

Biochemical detection of this type of cancer is nowadays restricted to CA 15–3. Its prognostic relevance is supported by a number of studies, but it was shown that it has insufficient utility (especially diagnostic sensitivity) at less advanced stages of BC [5, 6]. Hence, a search for new markers that would exhibit higher diagnostic performance is continuing. Due to the fact that angiogenesis and lymphangiogenesis are very important processes involved in the development of tumor changes and enable not only the creation of metastases, but also determine the local development of cancer [7], we predict that new candidates for tumor markers may be VEGF family members such as: VEGF-A, VEGF-C, VEGF-D and their receptor – VEGFR-2.

VEGF-A was discovered in 1989, its gene consists of 8 exons and plays an important role in the process of blood vessels forming [8, 9]. It is synthesized by various cell types, including mast cells, smooth muscle cells in vessels, macrophages, fibroblasts, cancer cells, endothelial cells, monocytes, keratinocytes, eosinophils and T lymphocytes [10].

VEGF-C was first identified in 1996 and is essential for embryonic development in the lymph vessel formation process. It is produced as a precursor protein that is activated by intracellular pro-protein convertases [11]. It reveals a mitogenic and protective role for both lymphatic and blood vessels. The clear expression of VEGF-C is found in the heart, placenta, muscles, ovaries, intestines and some cancers. This factor is also responsible for the increase in permeability and diameter of lymph vessels [12–14].

VEGF-D is expressed in the lungs, skin, heart, skeletal muscle, gastrointestinal tract and some cancers. Stimulates the growth and migration of endothelial cells. Like VEGF-C, VEGF-D participates in the lymphangiogenesis process [10, 15, 16]. The VEGF-D gene encodes 7 exons. VEGF-D maturation is similar to VEGF-C and occurs by protein cleavage in the N and C-terminal regions. Recent reports have shown that overexpression of VEGF-D induces tumor lymphangiogenesis and promotes lymphatic metastases in tumor models in mice [17].

There are three commonly known soluble receptors for VEGFs (VEGFR-1, VEGFR-2, VEGFR-3) found on the cell surface. Each of them has the possibility of binding selected factors belonging to the VEGF family on the basis of different affinities and selectivity [18, 19]. VEGFR-2 has a stronger tyrosine kinase activity than VEGFR-1 despite lower affinity for VEGF-A. There is growing evidence that VEGFR-2 is predominantly responsible for endothelial cell response to VEGF in both physiological and pathological conditions. VEGFR-2 stimulation promotes growth, migration and formation of endothelial cells and increases vascular permeability. Failure in the formation of blood vessels causes death in the embryonic stage in mice deficient in flk-1, indicating that VEGFR-2 plays an important role in the formation of the cardiovascular system during this stage of development. The anti-VEGFR-2 antibody inhibits primary and metastatic tumor growth in mouse models, indicating the key role of VEGFR-2 in tumor angiogenesis [20].

The aim of the present study was to investigate the diagnostic utility (sensitivity, specificity, predictive values of positive and negative test results) and power (ROC curve analysis) of the selected VEGF family members, their receptor, and a comparative tumor marker CA 15–3 in breast cancer detection. In this study, healthy volunteers and women with benign breast lesions constituted one control group, which provided a more accurate reflection of the current female population. The data obtained in this study may prove the usefulness of the analysed parameters (separately and together) in the detection of BC as a new diagnostic panel.

## Material and Methods

### Patients

Table 1 shows the tested groups. The study included 120 breast cancer patients (BC) diagnosed by the oncology group. The patients were treated in the Department of Oncology, Medical University, Bialystok, Poland. Tumor classification and staging were conducted in accordance with the International Union Against Cancer Tumor-Node-Metastasis (UICC-TNM) classification. Breast cancer histopathology was established in all cases by tissue biopsy of the mammary tumor or following surgery from tumor tissues (all patients with *adenocarcinoma ductale)*. The pretreatment staging procedures included: physical and blood examinations, mammography, mammary ultrasound scanning, breast core biopsies and chest X-rays.

**Table 1:** Characteristics of breast cancer patients and control groups: benign breast tumor and healthy women

Study group			Number of patients
Tested group	Breast cancer patients	*adenocarcinoma ductale*	120
	Median age (range)		58 (39–83)
	Tumor stage	I	38
		II	41
		III	20
		IV	21
	Menopausal status:		
	- premenopausal		21
	- postmenopausal		99
Control group	Benign breast tumor patients		28
		*adenoma*	10
		*fibroadenoma*	18
	Median age (range)		48 (36–71)
	Menopausal status:		
	- premenopausal		10
	- postmenopausal		18
	Healthy women		32
	Median age (range)		49 (33–73)
	Menopausal status:		
	- premenopausal		14
	- postmenopausal		18

In addition, radio isotopic bone scans, the examination of bone marrow aspirates, and brain and chest CT scans were performed when necessary. None of the patients had received chemo- or radiotherapy prior to blood sample collection.

The control groups included 60 patients: 28 with benign breast tumors (*adenoma, fibroadenoma*) and 32 healthy, untreated women who underwent mammary gland examination performed by a gynecologist prior to blood sample collection. In addition, mammary ultrasound scanning was performed in all cases. Benign breast tumor histopathology was established in all cases by tissue biopsy of the mammary tumor or after surgery.

For each of the patients qualified for the control group, the exclusion criteria such as: active infections and symptoms of an infection (both bacterial and viral), other comorbidities which can affect cytokine concentrations (respiratory diseases, digestive tract diseases) or systemic diseases such as lupus or rheumatoid arthritis, or collagenosis were applied.

### Biochemical Analyses

Venous blood samples were collected from each patient into a EDTA tube (S-Monovette, SARSTEDT, Germany), centrifuged 1000 x g for 15 min at 2-8 °C to obtain plasma samples and stored at –85 °C until assayed. The tested parameters were measured with the enzyme-linked immunosorbent assay (ELISA) (VEGF-A, VEGF-C, VEGF-D, VEGFR-2 - R&D Systems Inc., Minneapolis, MN, USA) and chemiluminescent microparticle immunoassay (CMIA) (CA 15–3 - Abbott, Chicago, IL, USA) according to the manufacturer’s protocols. In ELISA, according to the manufacturer’s protocols, duplicate samples were assessed for each standard, control, and sample.

The intra-assay coefficient of variation (CV%) of: CA 15–3 is reported to be 2.2% at a mean concentration of 27.0 U/mL, SD = 0.6; VEGF-A to be 4.5% at a mean concentration of 235 pg/mL, SD = 10.6; VEGF-C to be 3.5% at a mean concentration of 1543 pg/mL, SD = 54.2; VEGF-D to be 4.2% at a mean concentration of 970 pg/mL, SD = 40.9; VEGFR-2 to be 2.9% at a mean concentration of 2995 pg/mL, SD = 87.9.

The inter-assay coefficient of variation (CV%) of: CA 15–3 is reported to be 2.6% at a mean concentration of 27.0 U/ml, SD = 0.7; VEGF-A to be 7.0% at a mean concentration of 250 pg/mL, SD = 17.4; VEGF-C to be 7.2% at a mean concentration of 1540 pg/mL, SD = 110; VEGF-D to be 7.2% at a mean concentration of 956 pg/mL, SD = 68.5; VEGFR-2 to be 5.7% at a mean concentration of 2962 pg/mL, SD = 169.

The value of intra- and inter- assay CVs were calculated by the manufacturers and enclosed in the reagent kits. The assay does not exhibit cross-reactivity or interference with numerous human cytokines and other growth factors.

### Statistical Analysis

Statistical analysis was performed by STATISTICA 12.0 (StatSoft, Tulsa, OK, USA). The preliminary statistical analysis (using the Shapiro-Wilk test) revealed that the tested parameters and tumor marker levels did not follow normal distribution. Consequently, statistical analysis between the groups was performed by using the U-Mann Whitney test, the Kruskal-Wallis test and a multivariate analysis of various data by the post-hoc Dwass-Steele-Crichlow-Flinger test. The data were presented as a median and a range. Diagnostic sensitivity (SE), specificity (SP), and the predictive values of positive and negative test results (PPV and NPV, respectively) were calculated by using the *cut-off* values which were calculated by the Youden’s index (as a criterion for selecting the optimum *cut-off* point) and for each of the tested parameters were as follows: VEGF-A – 62.88 pg/mL; VEGF-C – 1552.85 pg/mL; VEGF-D – 562.15 pg/mL; VEGFR-2 – 8023.50 pg/mL; CA 15–3 – 18.45 U/mL. We also defined the receiver-operating characteristics (ROC) curve for all the tested parameters and tumor markers. The construction of the ROC curves was performed using the GraphRoc program for Windows (Windows, Royal, AR, USA) and the areas under the ROC curve (AUC) were calculated to evaluate the diagnostic accuracy and to compare AUC for all tested parameters separately and in combination with the commonly used tumor marker (CA 15–3). Statistically significant differences were defined as comparisons resulting in *p* < 0.05.

## Results

Table 2 shows the plasma levels of tested parameters and CA 15–3 in patients with breast cancer and in control groups. Plasma levels of VEGF-A, VEGF-C and CA 15–3 in total cancer group were statistically significantly higher when compared with total control group (in all cases p < 0.05). In divided control group (into benign breast tumor and healthy women group), we observed statistical significance when compared plasma levels of VEGF-A, VEGF-C and CA 15–3 with benign breast tumor and when compared VEGF-A, VEGF-C, VEGF-D and CA 15–3 with healthy women group.

**Table 2:** Plasma levels of tested parameters and CA 15–3 in patients with breast cancer and in control groups

Groups tested		VEGF-A (pg/mL)	VEGF-C (pg/mL)	VEGF-D (pg/mL)	VEGFR-2 (pg/mL)	CA 15–3 (U/mL)
Breast cancer Median Range	I stage	72.59 ^a/c^ 1.45–792.10	1941.75 ^a/b/c^ 432.22–4353.80	387.48 195.62–1584.30	8381.00 4051.50–12,118.50	16.70 ^a/c/d^ 6.20–50.30
	II stage	85.07 ^a/b/c^ 13.67–759.38	1860.55 ^a/b/c^ 905.50–4586.75	327.86 ^b/c^ 181.51–1981.10	8693.50 3066.30–12,937.00	16.90 ^a/b/c/d^ 4.40–48.10
	III stage	82.53 ^a/b/c^ 36.50–180.26	1788.25 ^a/c^ 1034.05–2528.90	376.64 246.55–560.93	8473.00 5717.00–11,073.00	26.50 ^a/b/c/d^ 8.90–167.50
	IV stage	98.00 ^a/b/c^ 21.60–251.66	1752.35 ^a/b/c^ 1286.40–2412.40	317.02 251.97–810.27	8237.50 6549.00–12,545.00	45.10 ^a/b/c/d^ 18.50–250.00
	Total group	78.50 ^a/b/c^ 1.45–792.10	1830.00 ^a/b/c^ 432.22–4586.75	346.83 ^b^ 181.51–1981.10	8523.25 3066.30–12,937.00	19.95 ^a/b/c^ 4.40–250.00
Control groups Median Range	Benign breast tumor	19.35 11.25–141.24	721.20^e^ 386.11–2581.00	439.87 217.57–1115.00	8005.00 6554.00–10,912.50	12.75 4.00–20.70
	Healthy women	46.79 7.51–197.36	1508.25^e^ 721.20–2849.10	413.80 258.74–1622.70	7947.50 5648.00–12,151.00	13.40 6.30–28.40
	Total group	31.36 7.51–197.36	1229.15 386.11–2849.10	413.80 217.57–1622.70	7959.00 5648.00–12,151.00	13.05 4.00–28.40

^a^Statistically significant when compared with benign breast tumor;

^b^Statistically significant when compared with healthy women;

^c^Statistically significant when compared with total control group;

^d^Statistically significant when BC patients stage III or IV compared with BC patients stage I or II;

^e^Statistically significant when compared healthy women with benign breast tumor

When compared to total control group, in I, III and IV stage of cancer VEGF-A, VEGF-C and CA 15–3, and in II stage VEGF-A, VEGF-C, VEGF-D and CA 15–3 showed statistical significance. When compared to benign breast tumor group, in all stages of cancer VEGF-A, VEGF-C and CA 15–3, showed statistical significance. When compared to healthy volunteers group, in I stage of cancer only VEGF-C, in II – VEGF-A, VEGF-C, VEGF-D and CA 15–3, in stage III – VEGF-A and CA 15–3, in stage IV – VEGF-A, VEGF-C and CA 15–3, showed statistical significance (in all cases *p* < 0.05).

VEGF-C was the only parameter, in which we have observed statistical significance in differentiation between benign breast tumor patients and healthy women group.

Table 3 shows the sensitivity (SE), specificity (SP), positive predictive value (PPV) and negative predictive value (NPV) of the investigated parameters and CA 15–3. We indicated that the SE of all tested parameters in the total cancer group was the highest for VEGF-D (82.50%). Among all parameters, the highest SE from tested parameters in stages I, II and III of cancer was observed also for VEGF-D (71.05%; 90.24%; 100%, respectively), in case of IV stage for CA 15–3 (90.48%). The diagnostic SP of the tested parameters was the highest for VEGF-A (76.67%), but lower than commonly used tumor marker (95%).

**Table 3:** Diagnostic criteria of tested parameters and CA 15–3 in patients with breast cancer

Tested parameters	Diagnostic criteria (%)	Breast cancer				
		I stage	II stage	III stage	IV stage	Total group
CA 15–3	SE SP PPV NPV	39.47 95.00 83.33 71.25	46.34 95.00 86.36 72.15	75.00 95.00 83.33 91.94	90.48 95.00 86.36 96.61	58.33 95.00 95.89 53.27
VEGF-A	SE SP PPV NPV	55.26 76.67 60.00 73.02	70.73 76.67 67.44 79.31	70.00 76.67 50.00 88.46	66.67 76.67 50.00 86.79	65.00 76.67 84.78 52.27
VEGF-C	SE SP PPV NPV	68.42 75.00 63.41 78.95	68.29 75.00 65.12 77.59	60.00 75.00 44.44 84.91	61.90 75.00 46.43 84.91	65.83 75.00 84.04 52.33
VEGF-D	SE SP PPV NPV	71.05 35.00 40.91 65.63	90.24 35.00 48.68 84.00	100.00 35.00 33.90 100.00	71.43 35.00 27.78 77.78	82.50 35.00 71.74 50.00
VEGFR-2	SE SP PPV NPV	52.63 53.33 41.67 64.00	65.85 53.33 49.09 69.57	65.00 53.33 31.71 82.05	71.43 53.33 34.88 84.21	62.50 53.33 72.82 41.56
CA 15–3 + VEGF-A	SE SP PPV NPV	71.05 73.33 62.79 80.00	78.05 73.33 66.67 83.02	100.00 73.33 55.55 100.00	100.00 73.33 56.68 100.00	83.33 73.33 86.21 68.75
CA 15–3 + VEGF-C	SE SP PPV NPV	78.95 70.00 62.50 84.00	82.93 70.00 65.38 85.71	95.00 70.00 51.35 97.67	100.00 70.00 53.85 100.00	86.67 70.00 85.25 72.41
CA 15–3 + VEGF-D	SE SP PPV NPV	81.58 35.00 44.29 75.00	92.68 35.00 49.35 87.50	100.00 35.00 33.90 100.00	100.00 35.00 35.00 100.00	91.67 35.00 73.83 67.74
CA 15–3 + VEGFR-2	SE SP PPV NPV	71.05 51.67 48.21 73.81	78.05 51.67 52.46 77.50	80.00 51.67 35.55 88.57	100.00 51.67 42.00 100.00	80.00 51.67 76.80 56.36

The predictive value of a positive test result (PPV) in the total group of BC patients was the highest for VEGF-A (84.78%), but lower than CA 15–3 (95.89%). Among all the tested parameters, the highest PPV values in I stage was observed for VEGF-C (63.41%), for stages II-IV of cancer were observed for VEGF-A (67.44%; 50%; 50%, respectively), but they were also lower than CA 15–3.

The predictive value of a negative test result (NPV) in the total group of BC was the highest for VEGF-C (52.33%), but was slightly lower than CA 15–3 (53.27%). The highest NPV in stage I of BC was observed for VEGF-C (78.95%), II and III – VEGF-D (84%), IV – CA 15–3 (96.61%).

Combined analysis of tested parameters and CA 15–3 resulted in increase of SE and NPV in almost all cases. The most favorable combination revealed to be CA 15–3 + VEGF-C and CA 15–3 + VEGF-A in total group of BC.

**Fig. 1:**
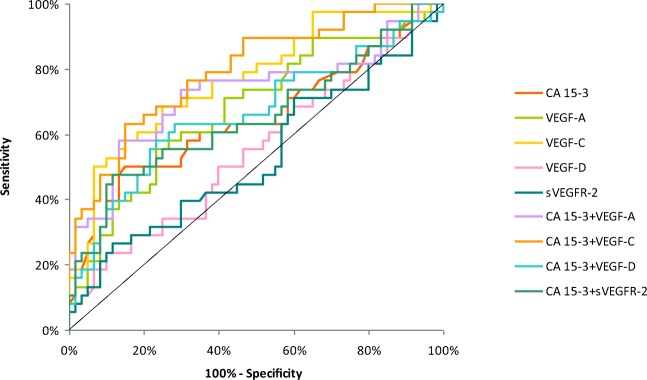
Diagnostic criteria of ROC curve for tested parameters and in combination with CA 15–3 in stage I of BC

**Fig. 2:**
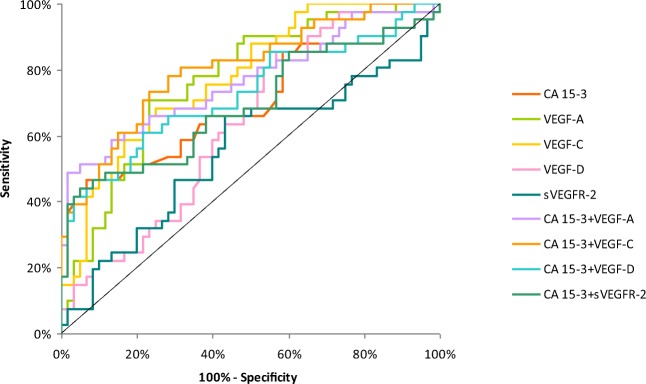
Diagnostic criteria of ROC curve for tested parameters and in combination with CA 15–3 in stage II of BC

**Fig. 3:**
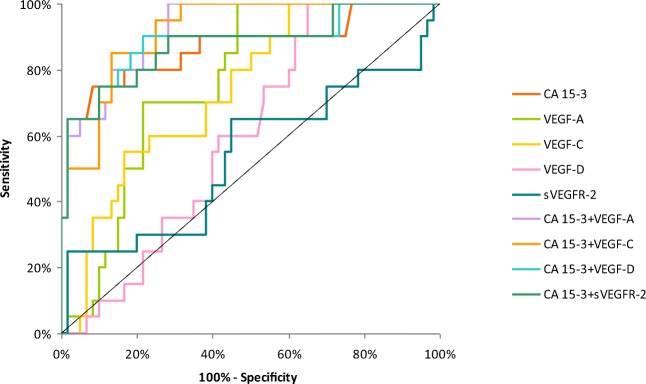
Diagnostic criteria of ROC curve for tested parameters and in combination with CA 15–3 in stage III of BC

**Fig. 4:**
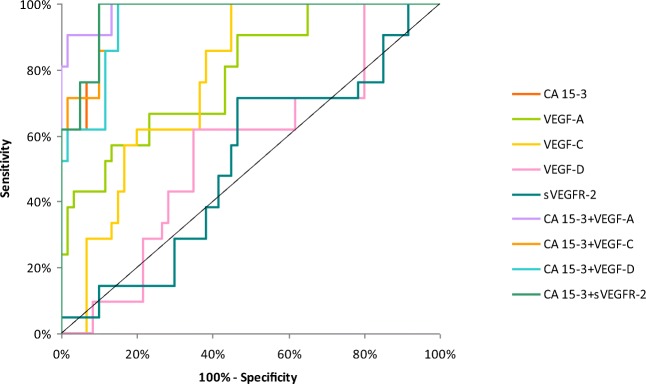
Diagnostic criteria of ROC curve for tested parameters and in combination with CA 15–3 in stage IV of BC

**Fig. 5:**
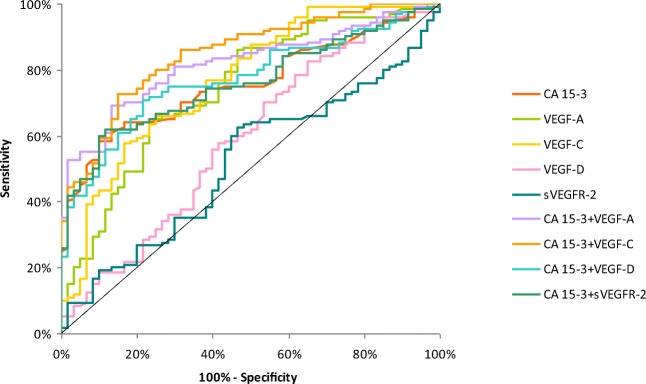
Diagnostic criteria of ROC curve for tested parameters and in combination with CA 15–3 in total BC group

The relationship between the diagnostic SE and SP is illustrated by the ROC curve. The area under the ROC curve (AUC) indicates the clinical usefulness of a tumor marker and its diagnostic power. All data relating to the AUC’s in total group of BC are included in Table 4. Graphical versions of the ROC curve for all tested parameters and their combinations with commonly used tumor marker in the whole group and all stages of BC are shown in Figs. 1, 2, 3, 4 and 5. We noticed that the VEGF-C area under the ROC curve (0.7672) in the total group of breast cancer was highest from all single tested parameters. In case of stages I and II of BC, AUC was highest also for VEGF-C (0.7684; 0.7772, respectively) from tested parameters, only in stages III and IV, AUC of CA 15–3 was higher (0.8692; 0.9667, respectively). Combined analysis of tested parameters and CA 15–3 resulted in increase of AUC in all cases. The most favorable combinations in total cancer group revealed to be VEGF-C and CA 15–3 (0.8476). The AUCs for the tested parameters, similarly as for commonly used tumor markers, were statistically significantly larger in comparison to AUC =0.5 (borderline of the diagnostic usefulness of the test) (*p* < 0.05 in all cases).

**Table 4:** Diagnostic criteria of ROC curve for tested parameters in all stages of BC

Tested parameters	AUC	SE	95% C.I. (AUC)	*p* (AUC = 0.5)
	ROC criteria in breast cancer (I stage)			
CA 15–3	0.6480	0.0610	(0.528–0.768)	0.0153
VEGF-A	0.6781	0.0571	(0.566–0.790)	0.0018
VEGF-C	0.7684	0.0498	(0.671–0.866)	<0.001
VEGF-D	0.5487	0.0613	(0.428–0.669)	0.4275
VEGFR-2	0.5287	0.0625	(0.406–0.651)	0.6456
CA 15–3 + VEGF-A	0.7232	0.0577	(0.610–0.836)	<0.001
CA 15–3 + VEGF-C	0.7956	0.0465	(0.704–0.887)	<0.001
CA 15–3 + VEGF-D	0.6623	0.0599	(0.545–0.780)	0.0067
CA 15–3 + VEGFR-2	0.6509	0.0606	(0.532–0.770)	0.0128
	ROC criteria in breast cancer (II stage)			
CA 15–3	0.6967	0.0567	(0.586–0.808)	<0.001
VEGF-A	0.7675	0.0473	(0.675–0.860)	<0.001
VEGF-C	0.7772	0.0457	(0.688–0.867)	<0.001
VEGF-D	0.6327	0.0554	(0.524–0.741)	0.0167
VEGFR-2	0.5528	0.0611	(0.433–0.673)	0.3875
CA 15–3 + VEGF-A	0.7663	0.0507	(0.667–0.866)	<0.001
CA 15–3 + VEGF-C	0.8033	0.0450	(0.715–0.891)	<0.001
CA 15–3 + VEGF-D	0.7268	0.0540	(0.621–0.833)	<0.001
CA 15–3 + VEGFR-2	0.6919	0.0570	(0.580–0.804)	<0.001
	ROC criteria in breast cancer (III stage)			
CA 15–3	0.8692	0.0555	(0.760–0.978)	<0.001
VEGF-A	0.7625	0.0532	(0.658–0.867)	<0.001
VEGF-C	0.7375	0.0590	(0.622–0.853)	<0.001
VEGF-D	0.6008	0.0637	(0.476–0.726)	0.1134
VEGFR-2	0.5375	0.0838	(0.373–0.702)	0.6546
CA 15–3 + VEGF-A	0.9242	0.0292	(0.867–0.981)	<0.001
CA 15–3 + VEGF-C	0.9167	0.0305	(0.857–0.976)	<0.001
CA 15–3 + VEGF-D	0.8842	0.0519	(0.782–0.986)	<0.001
CA 15–3 + VEGFR-2	0.8767	0.0520	(0.775–0.979)	<0.001
	ROC criteria in breast cancer (IV stage)			
CA 15–3	0.9667	0.0165	(0.934–0.999)	<0.001
VEGF-A	0.7881	0.0574	(0.676–0.901)	<0.001
VEGF-C	0.7738	0.0516	(0.673–0.875)	<0.001
VEGF-D	0.5571	0.0716	(0.417–0.697)	0.4246
VEGFR-2	0.5159	0.0732	(0.372–0.659)	0.8282
CA 15–3 + VEGF-A	0.9857	0.0100	(0.966–1.005)	<0.001
CA 15–3 + VEGF-C	0.9627	0.0177	(0.928–0.997)	<0.001
CA 15–3 + VEGF-D	0.9492	0.0217	(0.907–0.992)	<0.001
CA 15–3 + VEGFR-2	0.9690	0.0156	(0.938–1.000)	<0.001
	ROC criteria in total breast cancer group			
CA 15–3	0.7573	0.0351	(0.688–0.826)	<0.001
VEGF-A	0.7419	0.0395	(0.665–0.819)	<0.001
VEGF-C	0.7672	0.0381	(0.693–0.842)	<0.001
VEGF-D	0.5876	0.0464	(0.497–0.679)	0.0593
VEGFR-2	0.5180	0.0448	(0.430–0.606)	0.6881
CA 15–3 + VEGF-A	0.8174	0.0309	(0.757–0.878)	<0.001
CA 15–3 + VEGF-C	0.8476	0.0289	(0.791–0.904)	<0.001
CA 15–3 + VEGF-D	0.7715	0.0347	(0.704–0.839)	<0.001
CA 15–3 + VEGFR-2	0.7582	0.0350	(0.690–0.827)	<0.001

*p -* statistically significantly larger AUC’s compared to AUC = 0.5

Additionally, we have checked the correlations between tested parameters and lymph node metastasis, but no correlations has been shown.

## Discussion

Angio- and lymphangiogenesis are crucial for tumor progression and nutrition. Vascular endothelial growth factor family members and their receptors have a direct effect on endothelial cell proliferation, migration and are a potent stimulatory factors of those processes. Early diagnosis and determination of cancer stage allows to increase the survival rate of patients with breast cancer by indicating effective treatment methods. Due to many reports regarding the usefulness of tumor markers not only in breast cancer, it is very important that the diagnosis is not limited to diagnostic imaging [4, 6, 21, 22].

In the present study we investigated the usefulness of VEGF-A, VEGF-C, VEGF-D and VEGFR-2 separately and in combination with CA 15–3 (commonly used tumor marker) in breast cancer patients not only in the total group of patients but also in particular cancer stage groups (stages I, II, III and IV).

Statistically significant plasma over expression and high gene expression of VEGF-A, VEGF-C, VEGF-D and VEGFR-2 have been detected in patients suffering from many types of tumors, also breast cancer [6, 9, 23–27]. We have demonstrated statistically significantly higher plasma concentrations of almost all tested parameters when compared to control groups.

Comparable results for VEGF-A were obtained by Thielemann et al. [26] in breast cancer, but those authors compared their results only to healthy subjects group. In their research, cancer group consisted with stages I-III (TNM classification) of breast cancer. In their publication, both VEGF-A and VEGFR-2 revealed statistical significance in all stages of cancer when compared to healthy subjects. In our research, only VEGF-A showed statistical significance when compared to healthy subjects, in stages II-IV. This discrepancy might be related to different concentrations obtained in healthy controls group. In oppose to our findings were results obtained by Kotowicz et al. [27], where concentrations of VEGF-A and VEGFR-2 were not statistically significant, but their work concerned different type of tumor (endometrial cancer).

Comparable results (also statistically significant) for VEGF-C were obtained by Jensen et al. [28] in breast cancer-related lymphedema, but their results were about ten times lower than ours. In case of VEGF-D, in research commenced by Kummel et al. [29] in breast cancer patients plasma, mean concentrations were much lower than obtained by us (616 pg/mL vs. 98 ng/mL). This difference might be related to different composition of cancer group – their group consisted only from stages II and III (TNM classification).

Sensitivity (SE) measures the proportion of correctly identified positives. In this study, VEGF-D displayed the highest SE in the total group of breast cancer patients. To our knowledge, this work is first, which estimates not only concentrations but also diagnostic utility of VEGF-D. VEGF-A also revealed high SE (higher than commonly used tumor marker) not only in total cancer group, but also in stages I-IV. Results obtained by Kotowicz et al. [27] in I stage of endometrial cancer showed similar to ours SE in case of VEGF-A (56%), but much more lower SE in case of VEGFR-2 (18%). This discepancy might be caused by different type of examined tumor. We have found work contributed by Wu et al. [30] concerning pleural effusions, where VEGF-A was assesed as a prognostic factor. Their results were very promising and received high SE (76%), which shows that VEGF-A might be useful not only in cancer differentiation.

Specificity (SP) measures the proportion of correctly identified negatives. In this study, VEGF-A displayed the highest SP in the total control groups from tested parameters, but lower, than CA 15–3. We have obtained higher SP for VEGF-A and similar SP for CA 15–3 in our previous work in breast cancer [6], but in this work, we have used different method for calculating SP (95th percentile, not Youden index as now), which may be the reason for the differences in the ratio of diagnostic sensitivity and specificity. In work of Wu et al. [30], SP for VEGF-A was also high (84.2%).

Our results show that VEGF-A have the highest PPV values from all tested parameters in all groups of BC patients, but lower than commonly used tumor marker. As previously highlighted in our research, the PPV for VEGF-A was also very high [4, 6]. The predictive value of a negative test result (NPV) in the total group of BC and different stages of BC was mostly highest for VEGF-C, but lower than CA 15–3. Due our work is first to our knowledge, which contains not only concentrations, but also such a wide statistical analysis of VEGF-C cytokine, we are not able to compare our results to the work of other authors.

The most important criterion for tumor markers is the SE/SP diagram – ROC curve. The diagnostic power (AUC) represents the overall accuracy of a test, with the value approaching 1.0 indicating a perfect SE and SP. Our results showed that VEGF-C had the highest AUC of all the tested parameters in the total group of BC patients (0.7672) and stages I and II of this cancer. Comparable results (AUC = 0.803) for VEGF-C were obtained by Huang et al. [23] in papillary thyroid carcinoma.

In all diagnostic usefulness assessments, our research group is the only one, which evaluates the diagnostic usefulness of parameters in such a highly advanced way (combined analysis of all tested parameters with commonly used tumor marker). In this work, the best results were obtained by a combined analysis of CA 15–3 and VEGFR-3.

What is important, in future diagnosis, combined analysis of tested parameters with CA 15–3 can be the most correct way to improve the detection rate of breast cancer, because most of other parameters are non-specific and should be used only in panel to improve the sensitivity of the avaliable to date specific markers.

## Conclusions

Early detection of breast cancer in patients is of utter importance. Our present results indicate the usefulness and high diagnostic power of all the tested parameters in the detection of breast cancer. Among the tested parameters, VEGF-C appeared to be the best candidate for cancer diagnostics (superior to the commonly used tumor marker – CA 15–3) especially in stages I and II of BC. The combined analysis of the tested parameters and CA 15–3 resulted in an increase in SE and AUC values, which provides hope for developing a new panel of biomarkers that may be used in the diagnosis of BC in the future.

## Abbreviations

AUC: Area Under Curve
BC: breast cancer
NPV: negative predictive value
PPV: positive predictive value
ROC: Receiver Operating Characteristic
SE: sensitivity
SP: specificity
VEGF-A: vascular endothelial growth factor A
VEGF-C: vascular endothelial growth factor C
VEGF-D: vascular endothelial growth factor D
VEGFR-2: vascular endothelial growth factor receptor 2
